# Determination of Half-Maximal Inhibitory Concentration (IC_50_) of Drugs Using Contrast Surface Plasmon Imaging on Gold-Coated Periodic Nanowires

**DOI:** 10.1021/acs.analchem.5c02823

**Published:** 2025-09-16

**Authors:** Hsien-San Hou, Kuang-Li Lee, Ting-Jui Tu, Ji-Yen Cheng, Pei-Kuen Wei

**Affiliations:** † Research Center for Applied Sciences, 38017 Academia Sinica, Nankang, Taipei 11529, Taiwan; ‡ Biomedical Translation Research Center (BioTReC), Academia Sinica, Taipei 11529, Taiwan; § Institute of Biophotonics, National Yang Ming Chao Tung University, Taipei 11221, Taiwan; ∥ Department of Electrical Engineering, 59433 National Chi Nan University, Nantou County 545301, Taiwan

## Abstract

The half-maximal inhibitory concentration (IC_50_) is a critical pharmacological parameter used to quantify a drug’s potency in inhibiting biological functions. In this study, we present a novel strategy to evaluate the cytotoxicity of anticancer drugs on CL1-0 and A549 lung cancer cells, Huh-7 liver cancer cells, and MCF-7 breast cancer cells using contrast surface plasmon resonance (SPR) imaging with gold-coated periodic nanowire array sensors. The gold nanostructures, with a periodicity of 400 nm, produced a reflective SPR dip at 580 nm—positioned at the overlap between red and green channels of a color CCD sensor. The differential SPR response, captured through contrast imaging of red and green channels, reflected changes in cell adhesion. At predefined time points during cytotoxicity assessment, the attachment percentage of CL1-0 cells in response to doxorubicin treatment, along with the IC_50_ values for both CL1-0 and MCF-7 cells, was successfully quantified. Compared with conventional methods such as Cell Counting Kit-8 (CCK-8) and cell staining assays, the IC_50_ values derived from our SPR imaging platform aligned closely with those obtained via cell staining. Notably, CCK-8 failed to quantitatively assess the cytotoxic effect on MCF-7 cells, highlighting the limitations of enzymatic assays for certain cell types. Our innovative SPR imaging-based approach demonstrates that nanostructure-enhanced sensors enable accurate, high-throughput, and label-free IC_50_ determination for adherent cells. This platform offers a simple, low-cost alternative to traditional enzyme-dependent cytotoxicity assays.

## Graphical abstract

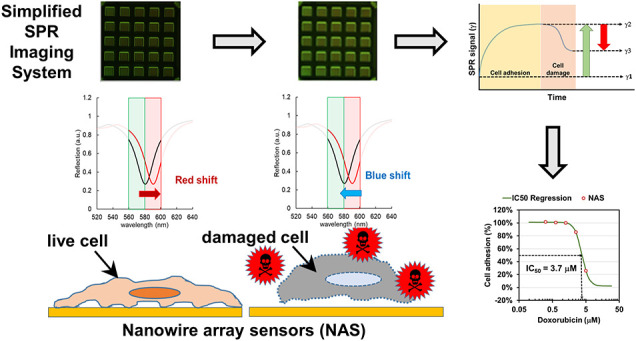

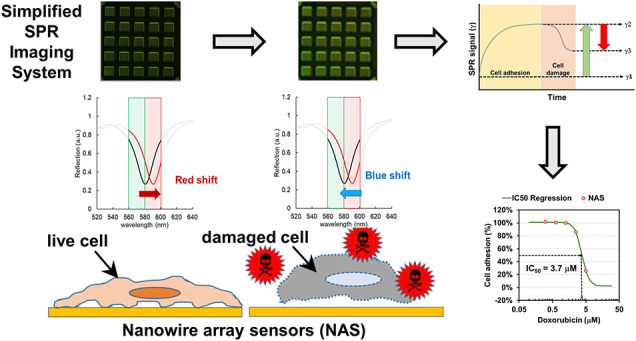

## Introduction

Cell viability assays are commonly used to evaluate the cytotoxic effects of compounds, toxins, or drugs. The half-maximal inhibitory concentration (IC_50_) is a key pharmacological parameter that quantifies a drug’s cytotoxic potency and serves as a benchmark for evaluating the efficacy of antitumor agents.^1^ In 2022, lung cancer and breast cancer were the most prevalent cancers among men and women worldwide, respectively. Therefore, evaluating the efficacy of anticancer drugs targeting these two types is crucial for advancing treatment strategies and improving patient outcomes.^2^ Traditional cell viability assays typically rely on the detection of intracellular dehydrogenase activity using tetrazolium salts such as MTT (3-[4,5-dimethylthiazol-2-yl]-2,5-diphenyl tetrazolium bromide) or Cell Counting Kit-8 (CCK-8),^3,4^ as well as fluorescence-based cell staining.^5^ These methods are classified as end-point assays, which capture data at fixed time intervals. As a result, critical temporal events, such as delayed toxicity or cellular recovery, may be missed. Moreover, reagents used in these assays can interfere with assay outcomes or damage the cells—e.g., reducing agents may affect MTT readouts—potentially compromising the accuracy of the results. End-point assays also provide only static measurements, limiting insight into the dynamic behavior of cells. Despite these limitations, such assays remain popular due to their simplicity, affordability, and accuracy at specific time points. Cell death, a fundamental biological process, occurs via two principal mechanisms: apoptosis and necrosis,^6^ both of which significantly impact cell morphology and adhesion. Apoptosis is characterized by controlled morphological changes, including cell shrinkage, chromatin condensation, and membrane blebbing, while maintaining plasma membrane integrity.^7^ It can be triggered by intrinsic factors such as DNA damage and oxidative stress or by extrinsic signals like death receptor activation. In contrast, necrosis is marked by uncontrolled cell swelling, membrane rupture, and cytoplasmic vacuolation,^7^ leading to a pronounced loss of cell adhesion. Because both apoptotic and necrotic processes induce alterations in cell attachment, monitoring changes in adhesion provides a valuable indicator of cell viability. Morphological and adhesion changes in adherent cells are frequently used as proxies for assessing cellular health.^8,9^

Over the past decade, real-time, label-free detection methods have emerged as powerful tools for investigating dynamic cellular processes. Techniques such as electric cell–substrate impedance sensing,^10^ resonant waveguide grating biosensors,^11^ and prism-based surface plasmon resonance (SPR)^12^ offer sensitive, real-time monitoring of cell behavior without the need for fluorescent labels or dyes. These noninvasive approaches avoid artifacts introduced by toxic or interfering reagents, preserve live cells for downstream analysis, and enable continuous observation of cellular responses. Among label-free detection techniques, SPR is a surface-sensitive optical method widely used to study molecular interactions—from small molecules to larger entities, such as bacteria and mammalian cells. It does not need to apply an external field. When integrated with imaging systems, SPR enables simultaneous detection across multiple sensing areas, making it highly suitable for multiple detections. However, despite these advantages, such systems are often costly, require complex instrumentation, and cannot be used for large-area detections, such as 96-well high-throughput screening applications.

In our previous studies, we demonstrated the use of metal nanostructure-based SPR chips to investigate cellular activities under external stimuli, such as cytotoxic drugs^13–15^ and shear stress.^16^ These nanostructured SPR chips were fabricated by using hot-embossing nanoimprint technology, enabling the generation of multiple SPR sensors on a single plastic substrate. This transmissive SPR platform is cost-effective, highly sensitive, and easy to manufacture. Unlike conventional prism-based SPR systems, which are typically limited to single or few detection channels, our multiwell SPR configuration allows for high-throughput analysis by capturing SPR spectra or images across multiple wells simultaneously.^17^ This system has been successfully applied to distinguish adhesion kinetics of primary vascular smooth muscle cells with and without Galectin-1 expression,^18^ as well as to monitor cellular responses under varying drug concentrations. In these applications, the transmission-mode SPR signal—commonly referred to as extraordinary optical transmission (EOT)—is generated through the coupling of localized plasmonic modes in nanocavities with propagating SPR modes on the grating surface. This coupling produces an asymmetric Fano resonance characterized by a distinct peak-dip profile. The associated evanescent tails exhibit variability between the peak and dip regions,^19–21^ complicating both intensity- and image-based SPR measurements. Consequently, this configuration alone may not support consistent or accurate comparisons of IC_50_ values for different substances.

To overcome these limitations, we employed injection molding technology to fabricate SPR chips with significantly improved uniformity compared with those produced by hot-embossing methods. Additionally, we adopted a simplified reflection-type SPR configuration, enabling the direct and stable detection of SPR dip signals. Within this framework, we introduced a novel imaging contrast technique that captures spectral shifts at the SPR dip, allowing for a more robust and quantitative analysis. To precisely evaluate the cytotoxicity of anticancer drugs, we developed a self-referencing strategy based on time-resolved SPR imaging. This method involves capturing SPR images at three critical stages: (1) during initial cell seeding, (2) immediately after drug administration, and (3) 24 h post-treatment. The resulting contrast images are processed to extract spectral changes corresponding to cell adhesion strength. By tracking variations in cell attachment over time, we construct IC_50_ curves to quantitatively determine cytotoxicity values. This strategy offers a simple, cost-effective, and reliable platform for drug screening, bridging the gap between label-free, real-time monitoring and the quantitative accuracy of traditional viability assays. It holds strong potential for high-throughput evaluation of the anticancer drug efficacy.

## Materials and Methods

### Fabrication of the 5 × 5 Gold-Coated Nanowire Array Sensors

A 5 × 5 array of 400 nm-period nanoridge structures, each with an area of 5 mm × 5 mm, was fabricated on a Ni–Co mold using electron beam lithography followed by electroplating^22^ as shown in Figure 1a. This mold, containing 25 nanoslit arrays, served as a master template for replication. Utilizing compression-injection molding, large-area plastic nanostructure chips were mass-produced on polycarbonate substrates (H3000R, Mitsubishi Engineering-Plastics Corporation) with high fidelity (Sumitomo Heavy Industries, Ltd.).^19^ The Ni–Co mold featured nanoslit dimensions of 110 nm in width, 60 nm in depth, and 400 nm in periodicity, resulting in periodic nanowire structures imprinted on the plastic surface, as seen in Figure 1b. A 50 nm-thick gold layer was subsequently deposited onto the nanowire arrays using electron-beam (E-gun) evaporation, forming the gold-coated nanowire array sensors (NASs). The schematic diagram of the nanostructural pattern of NAS is shown in Figure S1a. In aqueous environments, the NAS exhibited a dip resonance at a wavelength of 575 nm, characteristic of surface plasmon resonance for 400 nm-period structures (Figure 1c). The spectral image of the NAS analyzed by the hyperspectral imaging (HSI) system is shown in Figure S1b. All of the spectra show the same SPR dip resonant profiles with a variation less than 2% (Figure S1c). It confirms that such methods can produce high-quality and uniform SPR chips using the mold-injection method. The complete NAS biochip was assembled by stacking two layers of 0.4 mm-thick double-sided tape, a 2 mm-thick poly­(methyl methacrylate) (PMMA) layer, and the NAS substrate (Figure 1d). Prior to conducting cell adhesion experiments, the assembled NAS biochips were treated with oxygen plasma (200 W, 120 s) to achieve sterilization and enhance surface hydrophilicity, thereby promoting efficient cell attachment.

**Figure 1:**
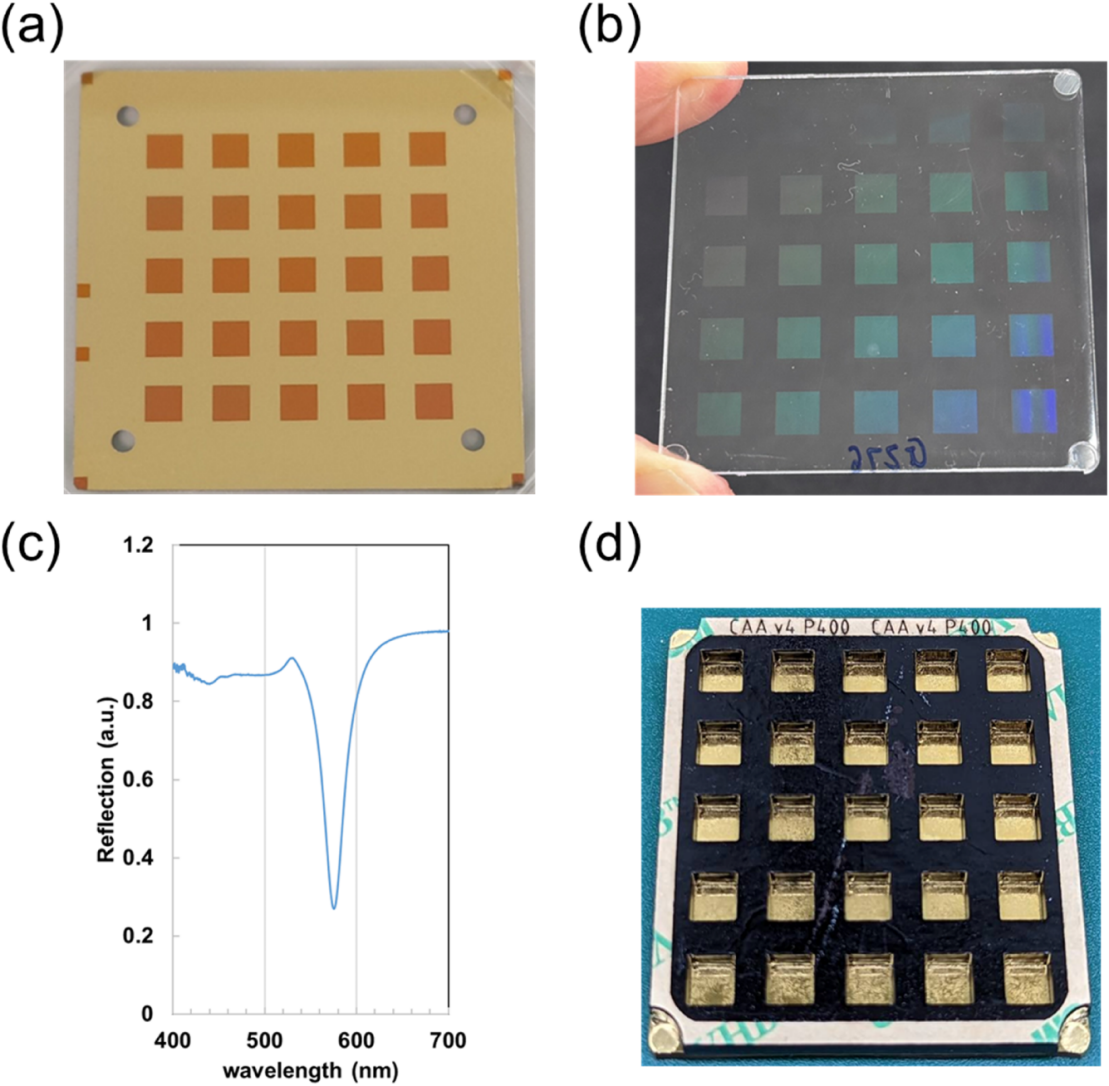
Fabrication of the NAS biochip for the cell adhesion assay. (a) Image of the Ni–Co mold used for large-scale NAS production. (b) Visualization of nanostructure arrays on a polycarbonate sheet fabricated through mold injection technology. (c) Reflective spectrum of the nanostructure pattern in water. (d) Image of the 25-well NAS biochip utilized for IC_50_ determination.

### Analysis of the Spectral Contrast Images

When the sCMOS camera captures RGB images of the NAS, each pixel is separated into three distinct color channels: red (R), green (G), and blue (B). The nanostructure of the NAS is specifically designed such that its SPR dip resonance aligns with the boundary region between the R and G channels, as illustrated in Figure 2a. To measure SPR shifts, a two-color spectral contrast analysis is employed, allowing for sensitive detection of SPR changes using the red and green channels.^20,21,23^ The γ value for a given region of interest is calculated using the following equation:

1 γ=((IG+δ)−(IR+δ))/(IG+IR+2δ)∼(IG−IR)/(IG+IR)

**Figure 2:**
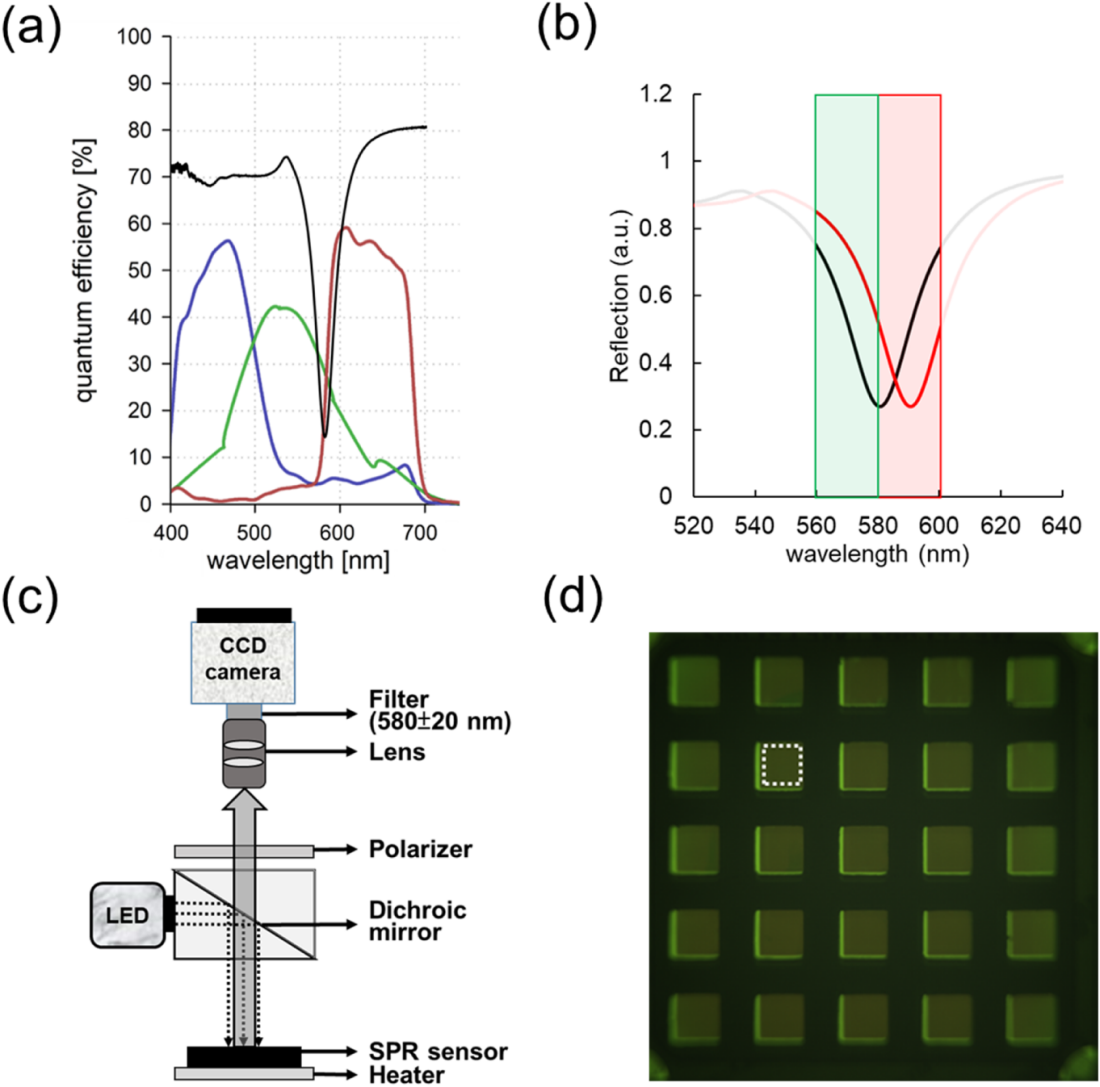
Cell adhesion assay utilizing the NAS chip. (a) Relevant spectra of the sCMOS camera and nanostructure sensor (black line) within the system. (b) Spectral shifts of NAS observed during the cell adhesion assay, with green and red bands representing the filter’s spectral range. (c) Schematic illustration of the simplified SPR imaging setup for the cell adhesion assay. (d) Contrast image of the NAS used for SPR signal analysis.

Here, IR and IG represent the intensity of the R channel and G channel in the image, respectively, and δ is a noise factor. Since this noise term cancels out in the calculation, the resulting measurement exhibits a very low noise floor—less than 10^–4^—thereby achieving a high signal-to-noise ratio in image-based SPR detection. However, when focusing on the SPR dip measurement, the region of interest corresponds to the lowest light intensity, as illustrated in Figure 2b. This poses a challenge, as the dominant intensity contributions from the R and G channels lie outside the SPR dip region. To address this limitation, a narrowband color filter centered at 580 nm is introduced to eliminate light outside the SPR-sensitive range. The bandwidth of the filter is critical, as the greatest intensity change during SPR shifts occurs near the half-maximum point of the resonance profile. In our NAS, the full width at half-maximum (fwhm) of the SPR dip is approximately 40 nm. Therefore, a bandpass filter with a center wavelength of 580 nm and a bandwidth of 40 nm is used to confine detection to the SPR-responsive region within the R and G channels. The reflection-mode SPR imaging system is illustrated in Figure 2c. It comprises a coaxial linear white LED light source, a bandpass filter (center wavelength = 580 nm, bandwidth = 40 nm), a polarizer, and an sCMOS camera (pco.panda 4.2c). The polarizer generates transverse magnetic (TM) polarized light to excite SPR on the surface of the NAS. The sCMOS camera captures red–green–blue (RGB) images, while the bandpass filter ensures that only relevant wavelengths reach the sensor, thereby enhancing detection sensitivity and contrast. The red and green channel images are converted into γ images using eq 1. Figure 2d displays the resulting gamma images from the 5 × 5 gold-coated NAS wells, captured simultaneously using the reflection-mode optical system. To monitor SPR responses over time, the gamma values for each of the 25 sensors were extracted, and their temporal changes were quantified by summing the gamma variation within each individual well.

### Cell Adhesion Procedure

Doxorubicin (DOX) and sorafenib were purchased from Sigma-Merck (Germany). The human lung cancer cell line CL1-0 was obtained from Prof. Pan-Chyr Yang.^24^ The cells were cultured in a complete medium composed of Dulbecco’s Modified Eagle’s Medium (DMEM, Gibco, USA) and 10% fetal bovine serum (FBS, Invitrogen, USA). The human breast cancer cell line MCF-7 and the hepatocyte-derived carcinoma cell line Huh-7 were obtained from Core Facilities of Translational Medicine of BioTReC (National Biotechnology Research Park, Academia Sinica). MCF-7 cells were cultured in a complete medium composing of Minimum Essential Medium (MEM, Sigma, USA) and 5% FBS. Huh-7 cells were cultured in a complete medium composing of low-glucose DMEM and 5% FBS. For regular maintenance, the cells were incubated at 37 °C with a 5% CO_2_ atmosphere and subcultured every 3 to 4 days. Cell adhesion experiments were performed with cells from the original source within 25 passages. Cells without mycoplasma infection were used in this study. Prior to introducing the cells into the NAS biochip, the culture wells were treated with a serum-free culture medium and incubated in a 5% CO_2_ incubator at 37 °C for 2 h to enhance cell adhesion. Approximately 85 μL of cells (5 × 10^5^ cells/mL) was suspended in a phenol-free medium and pipetted into the well of the NAS biochip. The biochip was then placed in the 5% CO_2_ cell culture incubator at 37 °C for further tests. The spectral contrast images of the NAS were captured at three specific time points: (1) immediately after cell seeding, (2) after overnight culture (approximately 18 h) to form a monolayer and immediately after adding drugs, and (3) after 24 h of coculture with drugs.

### Cell Viability Assessment

The cytotoxicity of the anticancer drug was tested using Cell Counting Kit-8 (CCK-8) and dead/total cell staining with PI/Hoechst fluorescent dyes. CCK-8 was purchased from Dojindo, Japan. The cell staining analysis was performed by Core Facilities of Translational Medicine of BioTReC. The cytotoxicity of the anticancer drug was plotted as cell viability versus drug concentration. IC_50_ values (half-maximal inhibitory concentration) were calculated using the online resource tool, MLA-“Quest Graph IC_50_ Calculator”—AAT Bioquest, Inc., https://www.aatbio.com/tools/ic50-calculator.^25^

## Results

### Optic System and Analytical Methods

When the SPR dip undergoes a red-shift due to an increased refractive index, the γ value rises. Conversely, a blue-shift of the SPR dip caused by a decreased refractive index results in a lower γ value. To enhance detection sensitivity, it is essential to align the SPR dip within the boundary region between the R and G channels of the camera’s quantum efficiency, ensuring that the γ value approaches zero. In Figure 1c, the resonance wavelength of the NAS in water is 575 nm, which falls at the crossing spectral point of G and R channels of the sCMOS camera, making it well-suited for use in spectral contrast imaging systems for analyzing cell behavior.^17^ The RGB image of the NAS with CL1-0 cells cultured inside the well is shown in Figure 2d. The dark green squares in the image are the area of nanoslit array structures used for sensing cell adhesion behaviors. A dashed square box, approximately 170 × 170 pixels in size, defines the region of interest (ROI) and is used to calculate the gamma value. The NAS biochip demonstrates a high-quality device with outstanding uniformity.

### Cell Adhesion and Refractive Index Variation

The kinetic changes in the SPR imaging (SPRi) signal related to cell adhesion and detachment using the NAS biochip are shown in Figure S2a. The initial status of the cell attachment, represented by γ_0_, defines the baseline of cell adhesion. After culturing the cells in the NAS biochip overnight, the γ value reached a plateau, referred to as γ_a_, indicating that the cells were fully spread and had completed the adhesion stage. Following the introduction of the drug to the cells for 24 h, cell injury and detachment occurred, and γ_d_ was used to define the detachment status. The cell adhesion level was calculated according to eq 2.

$$\mathrm{Cell}\;\mathrm{adhesion}\;\mathrm{level}\;\left(\%\right)=\left((\gamma_{d}-\gamma_{a})/(\gamma_{a}-\gamma_{0})\right)\times 100\%$$ 2

The dynamic changes in the NAS spectrum within the spectral contrast imaging system during cell adhesion evaluation are illustrated in Figure S2b. When cells adhere to the NAS surface, the increased contact area increases the refractive index, leading to a red shift in the SPR dip. In contrast, during cytotoxicity tests, drug-induced alterations reduce the contact area between the cells and the NAS, decreasing the refractive index and causing a blue shift in the SPR dip. These refractive index changes serve as an effective means of detecting cell adhesion behavior. The flowchart of the cytotoxicity assay is shown in Figure S2c.

### Characterization of Cell Adhesion Assessment using NAS Biochip

The effect of DOX on CL1-0 cells has been studied by using a real-time monitoring assay, but quantitative analysis of cell adhesion levels at varying DOX concentrations remains unexamined. Figure S2d illustrates changes in CL1-0 cell morphology during adhesion assessment after treatment with 10 μM DOX. Toxic concentrations of DOX resulted in cell death. The γ value, calculated using eq 1 from NAS images at specific time points, reached its maximum (γ = 0.21) 18 h post cell seeding (γ = 0.157). The introduction of DOX reduced cell attachment and the γ value (γ = 0.184). γ values at 0 h, 18 h, and DOX_24 h are denoted as γ_0_, γ_a_, and γ_d_, respectively. The cell adhesion level after 10 μM DOX treatment was quantified by using eq 2.

### Correlation of Cytotoxicity Assessment using CCK-8 and Cell Adhesion Assessment using NAS Biochip

The NAS biochip effectively evaluates the impact of DOX on CL1-0 cell adhesion, although its ability to assess cell viability via adhesion levels remains unverified. NAS and CCK-8 were used to examine the effects of DOX on the adhesion and viability of CL1-0 cells. Four to five independent experiments were conducted for both the CCK-8 assay and NAS test, with data on cell viability and adhesion percentages plotted against varying DOX concentrations. Figure 3a demonstrates a strong overlap between cell viability results from the CCK-8 and adhesion levels measured by NAS, highlighting a significant correlation between CL1-0 cell adhesion and viability in the presence of DOX. Statistical results from the CCK-8 assay and NAS biochip align closely. Fluorescent live/dead cell staining for DOX cytotoxicity testing on CL1-0 cells yielded viability results similar to those from CCK-8 and NAS tests, albeit with slight deviations (Figure 3b). This suggests that changes in adhesion levels can effectively represent CL1-0 cell viability.

**Figure 3:**
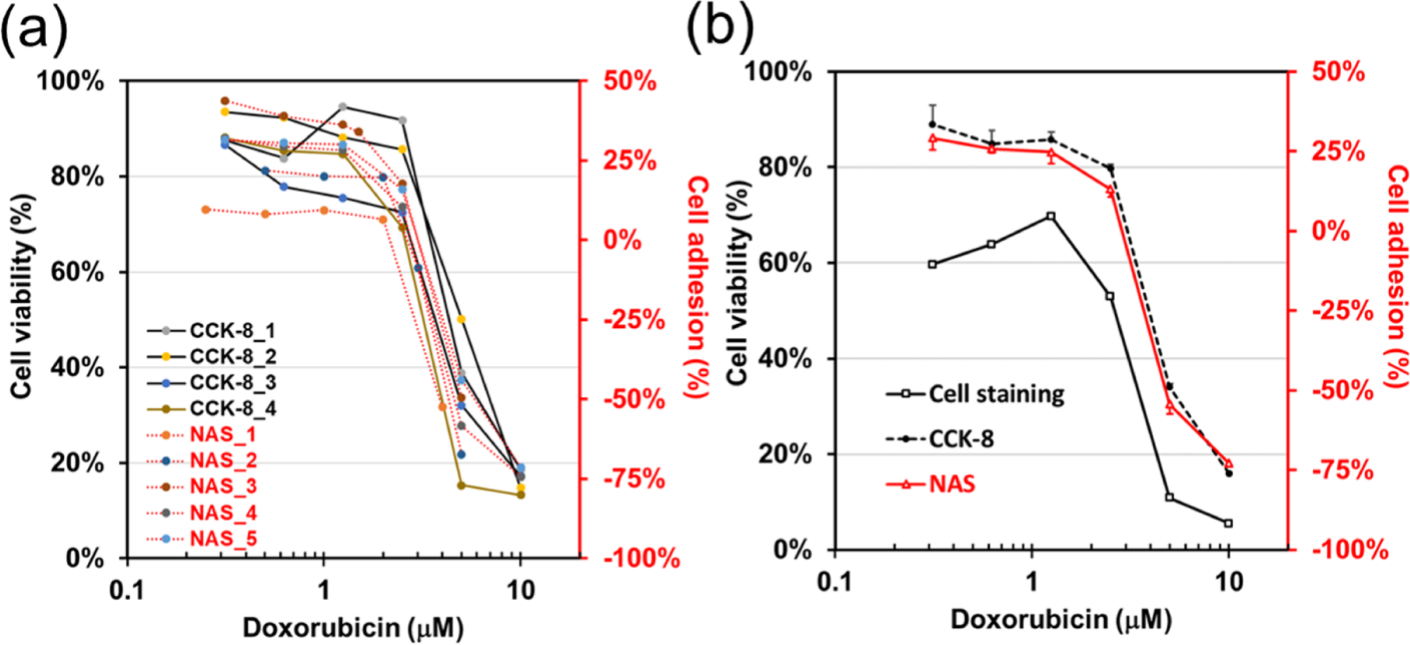
Effect of DOX on cell viability and adhesion tested by cell staining, CCK-8, and NAS in CL1-0 cells. (a) Results from CCK-8 and NAS were obtained in separate tests: cell viability was measured with CCK-8, and cell adhesion was quantified from eq 2 using the NAS biochip platform. (b) Statistical comparison of the three methods. Cell viability derived from the cell staining method was calculated using the following formula: viability (%) = [(total cells – dead cells)/total cells] × 100%. Cell adhesion and viability were used to compare results across three analytical approaches, enabling a quantitative assessment of consistency and accuracy among the NAS system, the CCK-8 assay, and cell staining.

### Quantitative Analysis of Cytotoxic Effects of Anticancer Drugs using CCK-8 and NAS

Cytotoxicity was evaluated after 24 h of treating CL1-0 cells with DOX. Cell viability and adhesion levels in response to the indicated DOX concentrations were assessed. However, comparing absolute cytotoxicity across methods was challenging due to the lack of numerical values. To address this, the IC_50_ value was employed as a quantitative measure for comparison between the CCK-8 and NAS biochip. IC_50_ values were calculated using the AAT Bioquest calculator with nonlinear regression curve fitting based on a variable slope.^25^ Regression curves for cell viability, derived from CCK-8 and cell staining, alongside cell adhesion from the NAS biochip, are presented in Figure 4a–c, respectively, showing similar slopes. The IC_50_ value of DOX in CL1-0 cells measured by the CCK-8 and NAS biochip was 3.11 ± 0.379 μM and 3.03 ± 0.445 μM, respectively, with no significant differences between methods. The experiment using the cell staining method produced an IC_50_ value of 3.24 μM, showing slight variation but sharing a similar slope compared to the other assays (Figure 4d). These findings confirm that cytotoxicity evaluation based on cell adhesion levels measured with the NAS biochip aligns with results from the CCK-8 or cell staining method. To evaluate the suitability of NAS for assessing the cytotoxicity of anticancer drugs across different cancer cell lines, we conducted comparative tests using sorafenib and DOX on Huh-7 liver cancer cells and A549 lung cancer cells. As illustrated in Figure S3a and b, the cytotoxic effects of sorafenib on Huh-7 cells were found to be similar when assessed using NAS and CCK-8. The IC_50_ values obtained were 10.7 ± 2.3 μM via NAS and 11.17 ± 1.92 μM via the CCK-8 assay, demonstrating no significant difference between the two methods (Figure S3c). Notably, drug resistance was observed in A549 cells at a DOX concentration of 10 μM when evaluated using the CCK-8 method, with the NAS approach producing comparable results, as illustrated in Figure S4. These findings confirm the suitability of NAS for assessing cell viability and highlight its applicability in the evaluation of various cell types. Part of the material has been used in 2024 IEEE SENSORS conference.^26^

**Figure 4:**
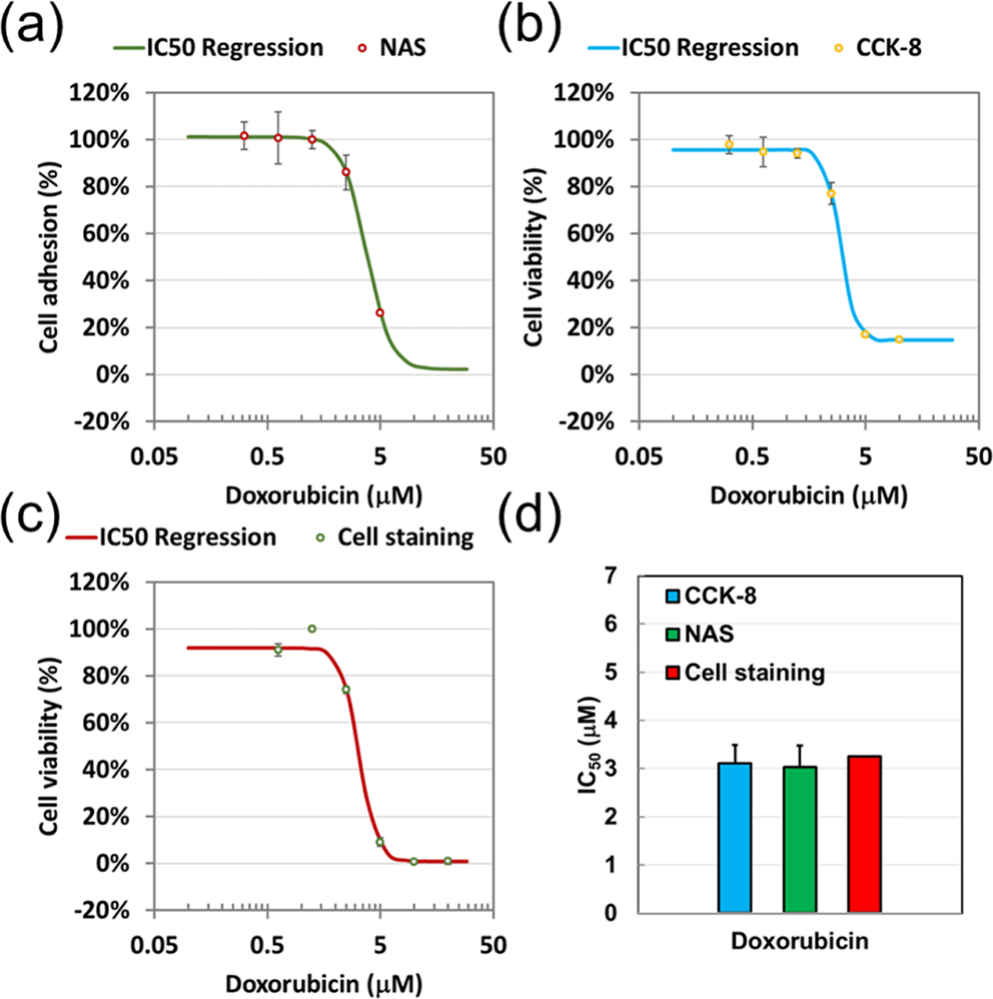
Effect of DOX on cell viability and adhesion converted by the IC50 calculator in CL1-0 cells. Cytotoxic effects of DOX on CL1-0 cells were assessed using three assays: (a) CCK8, (b) cell staining for cell viability, and (c) NAS for cell adhesion. All plotted and fitted data represent recalculated results obtained using the AAT Bioquest IC_50_ calculator. Nonlinear regression curve fitting with a variable slope (four-parameter model) was applied to analyze data from both viability and adhesion assays. (d) Statistical analysis summarizes IC_50_ values obtained from four independent experiments for each assay, providing a comparative assessment of DOX sensitivity across the different evaluation methods.

### The CCK-8 Assay Was Ineffective in Providing a Reliable Quantitative Analysis of DOX Cytotoxicity in MCF-7 Cells

In addition to lung and liver cancer cells, MCF-7 breast cancer cells were also utilized to assess the cytotoxicity of DOX using three different methods: CCK-8, NAS, and cell staining assays. MCF-7 cells of similar density were seeded in 96-well plates for the CCK-8 assay and in 25-well biochips for the NAS test. The regression curve derived from the cell adhesion analysis closely matched the cell viability results obtained through cell staining (Figure 5a and b). Interestingly, across three independent experiments, high doses of DOX resulted in increased cell viability in the CCK-8 assay, failing to accurately represent the cytotoxic effects of DOX on MCF-7 cells (Figure 5c). The IC_50_ value of DOX on MCF-7 cells, as measured by the NAS biochip, was 1.5 ± 0.124 μM—slightly lower than the 2.36 μM IC_50_ determined by the cell staining method (Figure 5d). These findings suggest that the NAS biochip’s measurement of cytotoxicity aligns well with the cell staining method but not with the CCK-8. Morphologically, in the cytotoxicity assessment, MCF-7 cells showed shrinkage and disruption of the cell monolayer at 1.25 and 2.5 μM of DOX treatment for the NAS biochip and CCK-8, respectively. This supports the hypothesis that the IC_50_ range of DOX on MCF-7 cells lies between 1.25 μM and 2.5 μM, consistent with results from the cell staining method. Figure 6 further shows the morphological differences of MCF-7 cells, as identified through the CCK-8 assay and the NAS biochip test. DOX-damaged MCF-7 cells appeared rounded in shape in the NAS biochip test (Figure 6a), whereas cell shrinkage was observed in the CCK-8 assay (Figure 6b). Whether the mechanisms of cell death differ between cultures in the NAS biochip and the 96-well plate remains a subject for further investigation.

**Figure 5:**
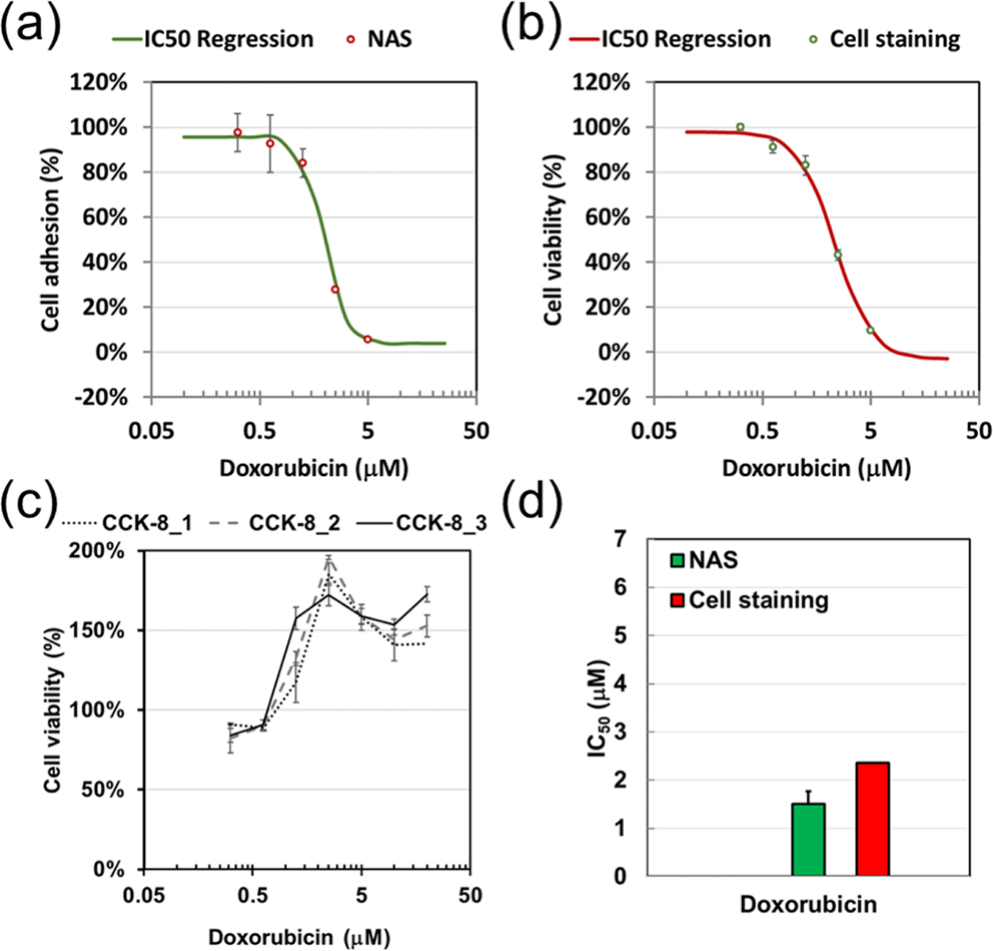
Effect of DOX on cell viability and adhesion in MCF-7 cells. The cytotoxic impact of DOX on MCF-7 cells was assessed using three assays: (a) NAS to evaluate cell adhesion, (b) cell staining, and (c) CCK-8 assay to determine viability. Data from the NAS and cell staining assays were recalculated, plotted, and fitted to determine the IC_50_ values using the AAT Bioquest IC_50_ calculator. Interestingly, CCK-8 assay results revealed that DOX did not significantly affect the viability of MCF-7 cells. (d) IC_50_ values were calculated from four independent experiments using NAS and cell staining data, providing a statistical comparison of DOX sensitivity based on adhesion-related responses.

**Figure 6:**
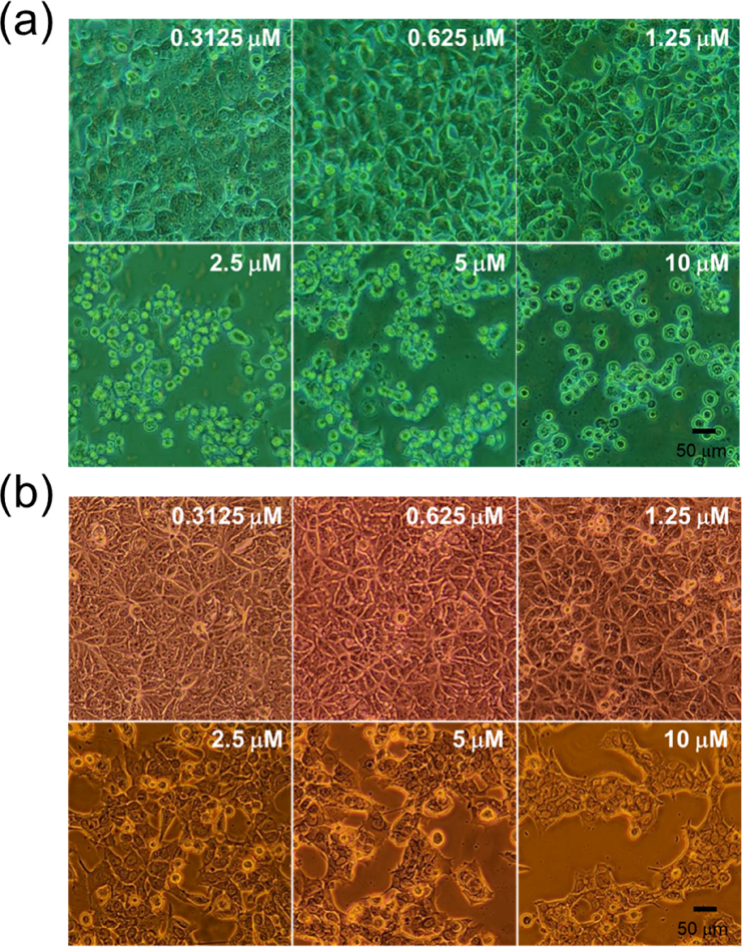
Effect of the cytotoxicity of DOX on MCF-7 cells. Cell morphology images of MCF-7 cells treated with DOX at indicated concentrations tested using (a) NAS and (b) CCK-8. In NAS and CCK-8 tests, at a concentration of 2.5 μM, significant changes in cell morphology occurred, and the intact cell monolayer was disrupted. In NAS assays, MCF-7 cells exhibited noticeable morphological changes—such as cell rounding and loss of spreading—at DOX concentrations above 2.5 μM, indicating pronounced cytotoxic effects. Similarly, in CCK-8 assays, reduced cell adhesion and spreading were observed at concentrations exceeding 2.5 μM, although the overall cytotoxicity appeared less severe compared to NAS results.

The anticancer activity of DOX is well-established and attributed to its ability to intercalate into DNA, inhibit topoisomerase II, disrupt mitochondrial function, and promote free radical generation and oxidative damage.^27^ Typically, DOX induces apoptotic cell death, with caspase-activated apoptosis occurring more rapidly (within a few hours)^28^ compared to DNA-damage-induced apoptosis (which may take dozens of hours).^29^ Interestingly, studies reveal that the cytotoxicity of citrus pectin, a compound causing DNA damage and subsequent cell death, is more pronounced at 72 h compared to 48 h when evaluated with the CCK-8 assay.^29^ This suggests that IC_50_ values may vary based on the timing of analysis, potentially due to differences in the rates of intracellular physiological disintegration after apoptosis is triggered. During early apoptosis, cells lose adhesion, which is evidenced by cell rounding and the redistribution of phosphatidylserine (PS) to the outer membrane leaflet.^30^ Subsequently, changes in cell morphology occur, including cytoplasmic shrinkage, membrane blebbing, and nuclear fragmentation. Membrane blebbing precedes the formation of apoptotic bodies, which package cellular contents into membrane-bound vesicles.^31^ Loss of membrane integrity, a late-stage event, follows PS exposure and morphological alterations. Propidium iodide (PI) uptake, used as a marker of permeability, is associated with DNA fragmentation and the late stages of apoptosis.^30^ Enzymatic activity, such as dehydrogenase function, declines as cellular metabolism slows during apoptosis. However, dehydrogenase levels may fluctuate throughout different stages of apoptosis, and residual enzymatic activity can persist in dead or damaged cells, potentially impacting assay sensitivity.^32^

In the cell staining method, where MCF-7 cells were cultured in 96-well plates like those used for the CCK-8 assay, IC_50_ values differed. This discrepancy is likely due to differences in detection indicators—cell membrane permeability in cell staining versus dehydrogenase levels in the CCK-8 assay. As the cell membrane permeability and specific enzyme content are viability indicators, the IC_50_ results from these two methods may not align. The CCK-8 test, however, requires careful optimization through pilot tests to determine the most suitable cell density, a process that demands operational experience. This highlights the practicality and reliability of the NAS biochip, which emerges as a more suitable platform for cytotoxicity testing.

## Conclusion

This study validated the correlation between cell viability, as measured by CCK-8 and cell staining, and cell adhesion assessed using the NAS biochip. The results demonstrated that cell adhesion measurements obtained with the NAS biochip are consistent with cell viability assays derived from conventional approaches, such as those utilizing CL1-0 cells. This suggests that changes in cell morphology, like those observed in the NAS biochip assessments, can serve as unique indicators of cell viability. Similar to how cell membrane permeability is evaluated in cell staining and intracellular hydrogenase levels in CCK-8, cell adhesion provides another reliable parameter for viability testing.

The effect of DOX on the adhesion of CL1-0 cells was quantified using a NAS biochip, revealing its cytotoxicity through IC_50_ value determination. Interestingly, the IC_50_ values obtained using CCK-8, cell staining, and the NAS biochip were identical, confirming that the NAS biochip is a viable tool for assessing cell viability based on adhesion. Furthermore, the NAS biochip supports the growth of various cell lines, including lung cancer cells, melanoma cells, kidney cells, breast cancer cells, and hepatocellular carcinoma cells. Notably, cells can survive on the NAS biochip for 2–3 days, making it a versatile platform for cytotoxicity studies.^15^

The NAS biochip offers a straightforward and user-friendly procedure for cell adhesion assessment. By capturing three sets of images, cell adhesion levels can be calculated using specialized software. Real-time monitoring is also possible through continuous image capture. Additionally, since reference images are used in calculations, there is no need for a separate reference well, further simplifying the process. The label-free nature of the NAS biochip-based assessment also reduces costs and saves time, enhancing its practicality. Table S1 presents a comparison of three cell viability assays—NAS platform, CCK-8, and cell staining—demonstrating that the NAS platform offers the most cost-effective approach for cytotoxicity assessment.

The SPR imaging system utilized in this study introduces significant improvements over traditional SPR systems by employing a fixed design that eliminates the need for movable components, precise alignment, and complex mechanical structures. This allows the NAS biochip to be used for discontinuous measurements at specific time points without significant errors. Measurements require the capture of cell images only at three intervals, negating the need for additional reagents. The biochip format is compatible with standard cell biology instruments and conventional well plates, ensuring easy integration into current workflows. During drug cytotoxicity testing, the entire cell culture process is conducted in a conventional incubator, reducing artificial impacts on the cells.

Compared with transmissive SPRi platforms, the reflective SPRi platform used in this study minimizes the effect of sensor defects on detection. Its reflective design allows light to penetrate the cell layer and nanogold layers before being reflected back to the camera, ensuring reliable detection. Defects in nanogold layers constructed through plastic injection molding, which can reduce sensitivity in transmissive systems, have less impact on the reflective platform.

This study represents the first quantitative determination of IC_50_ of anticancer drugs using a SPR imaging-based method. A comparison between the SPR-based cytotoxicity technique and previously reported methods for IC_50_ determination using electrical or optical sensing approaches is summarized in Table S2. Unlike other approaches, such as electrical impedance methods or traditional SPR spectroscopy, which monitor changes in cell monolayer integrity or cell attachment continuously, the NAS biochip allows precise, label-free cytotoxicity assessment. This innovative method significantly enhances the efficiency and accuracy of studying anticancer drugs’ effects on cancer cell lines, paving the way for more timely and precise analyses of drug efficacy and toxicity.

## Supplementary Material



## Notes

Results of the characterization of metallic nanostructures, detailed methodologies for cell viability assessment, and the cytotoxicity of sorafenib and doxorubicin on Huh-7 and A549 cell lines tested by a NAS biochip and the CCK-8 method (PDF)

## References

[1] Huang M. -C., Ashmun R. A., Avery T. L., Kuehl M., Blakley R. L. (1986). Effects
of cytotoxicity of 2-chloro-2’-deoxyadenosine and 2-bromo-2’-deoxyadenosine
on cell growth, clonogenicity, DNA synthesis, and cell cycle kinetics. Cancer Res.

[2] Bray F., Laversanne M., Sung H., Ferlay J., Siegel R. L., Soerjomataram I., Jemal A. (2024). Global cancer statistics 2022: GLOBOCAN
estimates of incidence and mortality worldwide for 36 cancers in 185
countries. Ca-Cancer J. Clin..

[3] Mosmann T. (1983). Rapid colorimetric
assay for cellular growth and survival: Application to proliferation
and cytotoxicity assays. J. Immunol. Methods.

[4] Berridge M. V., Herst P. M., Tan A. S. (2005). Tetrazolium dyes
as tools in cell
biology: New insights into their cellular reduction. Biotechnol. Annu. Rev..

[5] Nociari M. M., Shalev A., Benias P., Russo C. (1998). A novel one-step,
highly
sensitive fluorometric assay to evaluate cell-mediated cytotoxicity. J. Immunol. Methods.

[6] Fawthrop D. J., Boobis A. R., Davies D. S. (1991). Mechanisms
of cell death. Arch. Toxicol..

[7] Doonan F., Cotter T. G. (2008). Morphological assessment
of apoptosis. Methods.

[8] Kroemer G., Galluzzi L., Vandenabeele P., Abrams J., Alnemri E. S., Baehrecke E. H., Blagosklonny M. V., El-Deiry W. S., Golstein P., Green D. R. (2009). Classification of cell death: Recommendations
of the Nomenclature Committee on Cell Death 2009. Cell Death Differ..

[9] Ramsden J. J., Li S. Y., Prenosil J. E., Heinzle E. (1994). Kinetics of adhesion
and spreading of animal cells. Biotechnol. Bioeng..

[10] Wegener J., Keese C. R., Giaever I. (2000). Electric cell-substrate impedance
sensing (ECIS) as a noninvasive means to monitor the kinetics of cell
spreading to artificial surfaces. Exp. Cell
Res..

[11] Fang Y., Ferrie A. M., Fontaine N. H., Mauro J., Balakrishnan J. (2006). Resonant waveguide
grating biosensor for living cell sensing. Biophys.
J..

[12] van
der Merwe P. A., Barclay A. N. (1996). Analysis of cell-adhesion molecule
interactions using surface plasmon resonance. Curr. Opin. Immunol..

[13] Wu S. H., Nunez D., Hu S. Y., Domingo M. P., Chen Y. C., Wei P. K., Pardo J., Galvez E. M., Chiou A. (2014). The effect
of acidic pH on the inhibitory efficacy of peptides against the interaction
ICAM-1/LFA-1 studied by surface plasmon resonance (SPR). Biosens. Bioelectron..

[14] Wu S. H., Lee K. L., Chiou A., Cheng X., Wei P. K. (2013). Optofluidic
platform for real-time monitoring of live cell secretory activities
using Fano resonance in gold nanoslits. Small.

[15] Hou H. S., Lee K. L., Wang C. H., Hsieh T. H., Sun J. J., Wei P. K., Cheng J. Y. (2019). Simultaneous assessment of cell morphology
and adhesion using aluminum nanoslit-based plasmonic biosensing chips. Sci. Rep..

[16] Wu S. H., Lee K. L., Weng R. H., Zheng Z. X., Chiou A., Wei P. K. (2014). Dynamic monitoring
of mechano-sensing of cells by gold
nanoslit surface plasmon resonance sensor. PLoS
One.

[17] Lee K. L., Hou H. S., Cheng J. Y., Wei P. K. (2020). High-Throughput
and Dynamic Study of Drug and Cell Interactions Using Contrast Images
in Aluminum-Based Nanoslit Arrays. Anal. Chem..

[18] Tsai M. -S., Chiang M. -T., Tsai D. -L., Yang C. -W., Hou H. -S., Li Y. -R., Chang P. -C., Lin H. -H., Chen H. -Y., Hwang I. -S. (2018). Galectin-1 Restricts Vascular Smooth Muscle
Cell Motility Via Modulating Adhesion Force and Focal Adhesion Dynamics. Sci. Rep..

[19] Lee K.-L., You M.-L., Shi X., Li Y.-R., Ueno K., Misawa H., Wei P.-K. (2019). Injection
compression molding of
transmission-type Fano resonance biochips for multiplex sensing applications. Appl. Mater. Today.

[20] Pan M. Y., Yang D. K., Lai C. Y., Weng J. H., Lee K. L., Chen L. C., Chou C. F., Wei P. K. (2019). Spectral
contrast
imaging method for mapping transmission surface plasmon images in
metallic nanostructures. Biosens. Bioelectron..

[21] Lo S. C., Yeh C. W., Wang S. H., Kuo C. W., Lee K. L., Chern R. L., Wei P. K. (2021). Self-referencing
biosensors using
Fano resonance in periodic aluminium nanostructures. Nanoscale.

[22] Lee K. L., Wu T. Y., Hsu H. Y., Yang S. Y., Wei P. K. (2017). Low-Cost
and Rapid Fabrication of Metallic Nanostructures for Sensitive Biosensors
Using Hot-Embossing and Dielectric-Heating Nanoimprint Methods. Sensors.

[23] Lee K.-L., Hou H.-S., Shi X., You M.-L., Pan M.-Y., Matsuo Y., Cheng J.-Y., Misawa H., Wei P.-K. (2024). Aluminum-Coated
Nanoridge Arrays with Dual Evanescent Wavelengths for Real-Time and
Label-Free Cellular Analysis. J. Phys. Chem.
C.

[24] Chu Y. W., Yang P. C., Yang S. C., Shyu Y. C., Hendrix M. J., Wu R., Wu C. W. (1997). Selection of invasive
and metastatic subpopulations
from a human lung adenocarcinoma cell line. Am. J. Respir. Cell Mol. Biol..

[25] AAT Bioquest, Inc.. IC50 Calculator, 2025. https://www.aatbio.com/tools/ic50-calculator.

[26] Hou, H. S. ; Tu, T. J. ; Cheng, J. Y. ; Lee, K. L. ; Wei, P. K. Cytotoxicity Anlysis of Drugs Using Contrast Surface Plasmon Images in Gold Nanoslit Arrays 2024 IEEE SENSORS IEEE 2024 1–4 10.1109/SENSORS60989.2024.10784734

[27] Kciuk M., Gielecinska A., Mujwar S., Kolat D., Kaluzinska-Kolat Z., Celik I., Kontek R. (2023). Doxorubicin-An Agent
with Multiple
Mechanisms of Anticancer Activity. Cells.

[28] Kwon H.-K., Lee J.-H., Shin H.-J., Kim J.-H., Choi S. (2015). Structural
and functional analysis of cell adhesion and nuclear envelope nano-topography
in cell death. Sci. Rep..

[29] Salehi F., Behboudi H., Kavoosi G., Ardestani S. K. (2018). Oxidative
DNA damage induced by ROS-modulating agents with the ability to target
DNA: A comparison of the biological characteristics of citrus pectin
and apple pectin. Sci. Rep..

[30] Bailey R. W., Nguyen T., Robertson L., Gibbons E., Nelson J., Christensen R. E., Bell J. P., Judd A. M., Bell J. D. (2009). Sequence
of physical changes to the cell membrane during glucocorticoid-induced
apoptosis in S49 lymphoma cells. Biophys. J..

[31] Elmore S. (2007). Apoptosis:
A review of programmed cell death. Toxicol.
Pathol..

[32] Maehara Y., Anai H., Tamada R., Sugimachi K. (1987). The ATP assay
is more sensitive than the succinate dehydrogenase inhibition test
for predicting cell viability. Eur. J. Cancer
Clin. Oncol..

