# Supplementary material for: Determination of Half-Maximal Inhibitory Concentration (IC50) of Drugs Using Contrast Surface Plasmon Imaging on Gold-Coated Periodic Nanowires
Source: Anal Chem. 2025 Sep 16;97(38):20817–24. doi: 10.1021/acs.analchem.5c02823 (PMC12489899; doi:10.1021/acs.analchem.5c02823)
Supplement: Supplementary file 1 [file ac5c02823_si_001.pdf]

Supporting Information

Determination of Half-Maximal Inhibitory Concentration (IC_{50}) of Drugs Using Contrast Surface Plasmon Imaging on Gold-Coated Periodic Nanowires

*Hsien-San Hou[#1], Kuang-Li Lee[#4], Ting-Jui Tu[1], Ji-Yen Cheng[1,3], and Pei-Kuen Wei[1,2,3]**

1. Research Center for Applied Sciences, Academia Sinica, Nankang, Taipei 11529, Taiwan
2. Biomedical Translation Research Center (BioTReC), Academia Sinica, Taipei, 11529, Taiwan.
3. Institute of Biophotonics, National Yang Ming Chao Tung University, Taipei 11221, Taiwan
4. Department of Electrical Engineering, National Chi Nan University, Nantou County 545301, Taiwan

Author e-mail address: pkwei@sinica.edu.tw

[#]Contributed equally.

Table of contents

Figure S1. Characterization of NAS chip.

Figure S2. Approach for cytotoxicity determination using NAS chip.

Figure S3. Effect of Sorafenib on cell viability and adhesion in Huh-7 cells.

Figure S4. Effect of DOX on cell viability and adhesion assessed using NAS and CCK-8 in A549 cells.

Table S1. Comparison of cell viability assays.

Table S2. A comparative analysis of the SPR-based cytotoxicity assessment technique with established electrical and optical sensing methods for evaluating cytotoxicity and determining IC_{50} values.

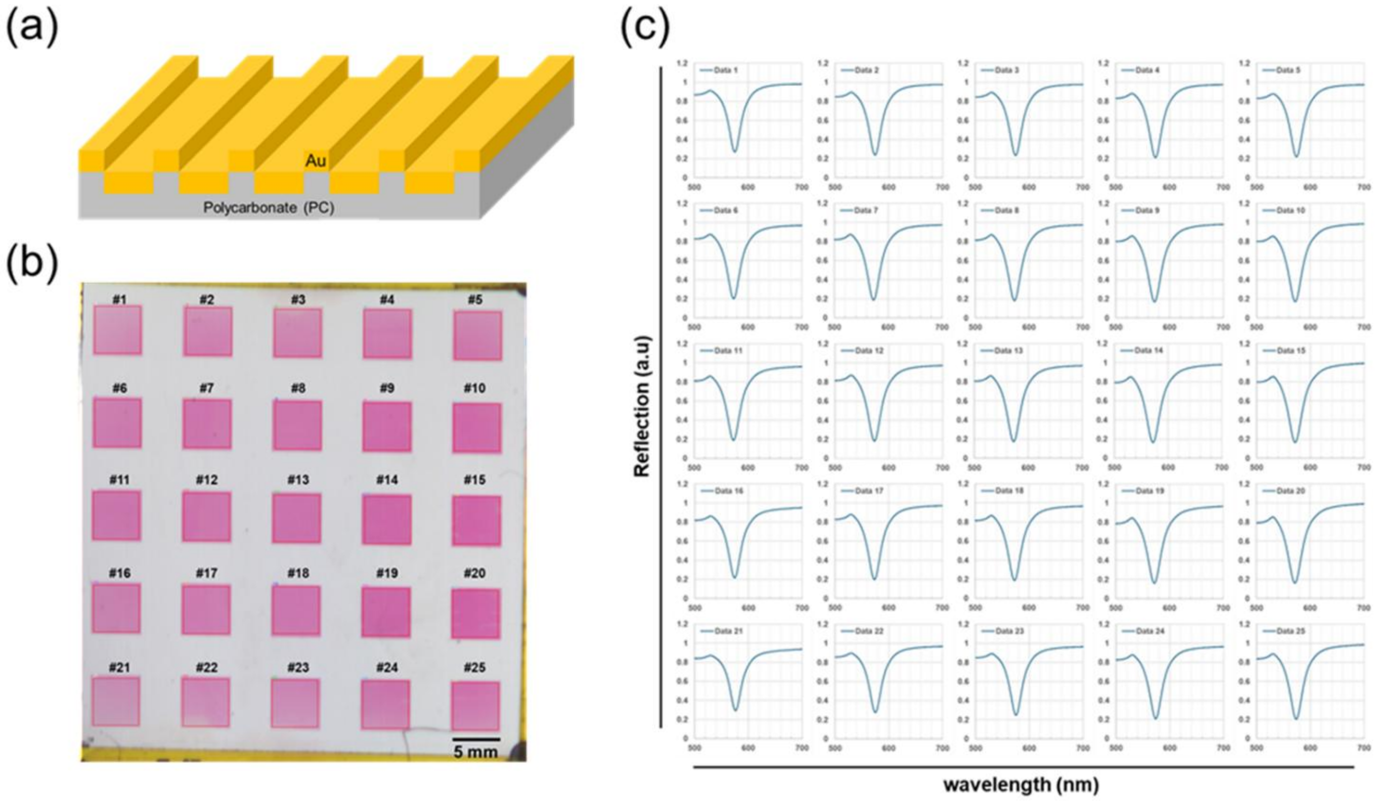

Figure S1. (a) Schematic representation of the NAS nanostructure in this study. (b) NAS spectral image analyzed using a hyperspectral imaging system, showing a uniform and well-defined nanostructure pattern. (c) Spectral analysis of 25 nanostructural patterns on the NAS.

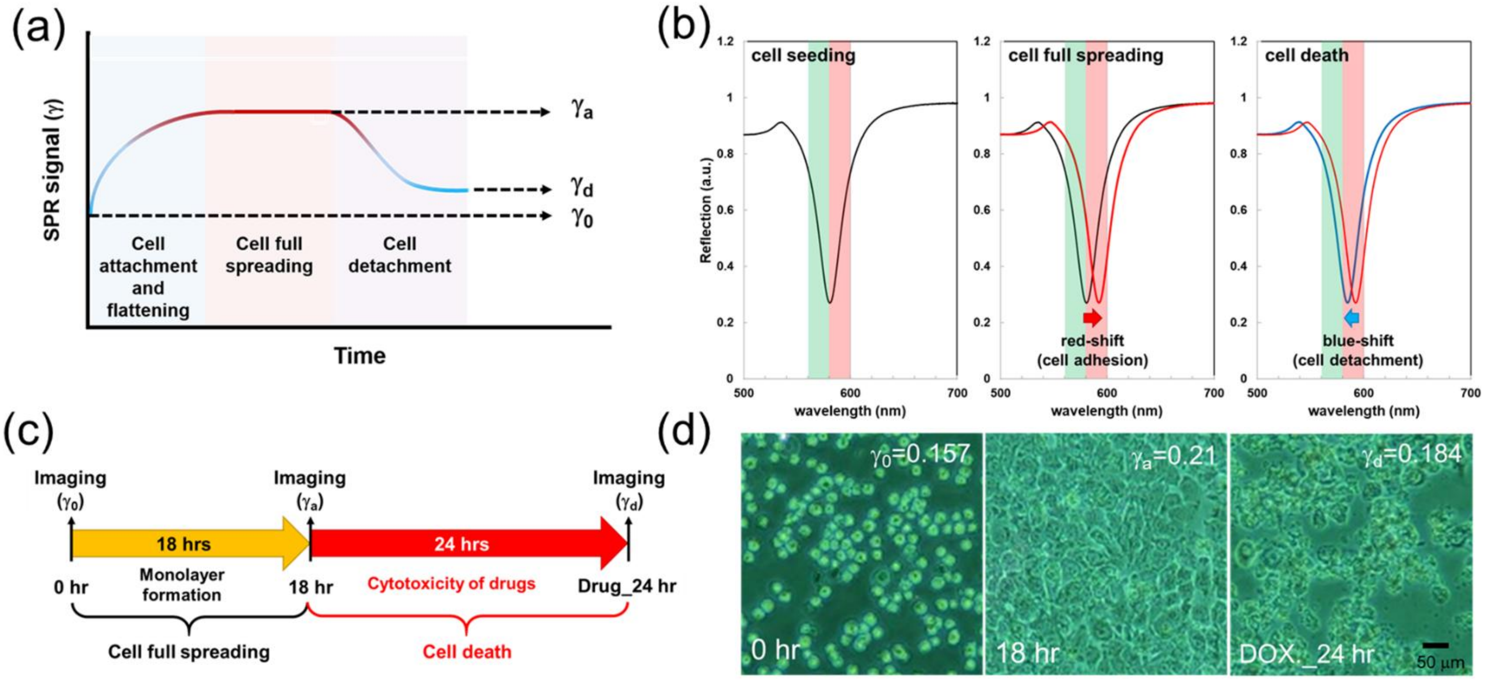

Figure S2. Approach for cytotoxicity determination using NAS chip. (a) Schematic of dynamic changes of SPRi signal during cytotoxicity evaluation. (b) Spectral shifts of the NAS sensor during cell seeding, spreading, and death. During the stages of cell seeding, full spreading, and cell death, the NAS sensor exhibited distinct spectral shifts. The green and red bands indicate the filter's spectral detection range. As cells spread and increase contact with the sensor surface, a red-shift in the spectrum is observed due to enhanced mass loading. Conversely, drug-induced reduction in cell adhesion decreases surface contact, resulting in a spectral blue-shift. (c) Schematic diagram of the procedure of the test. (d) The cellular morphology photo of CL1-0 cells culturing in the NAS during cytotoxicity assessment. The gamma value in the photo is calculated from the NAS images at indicated time.

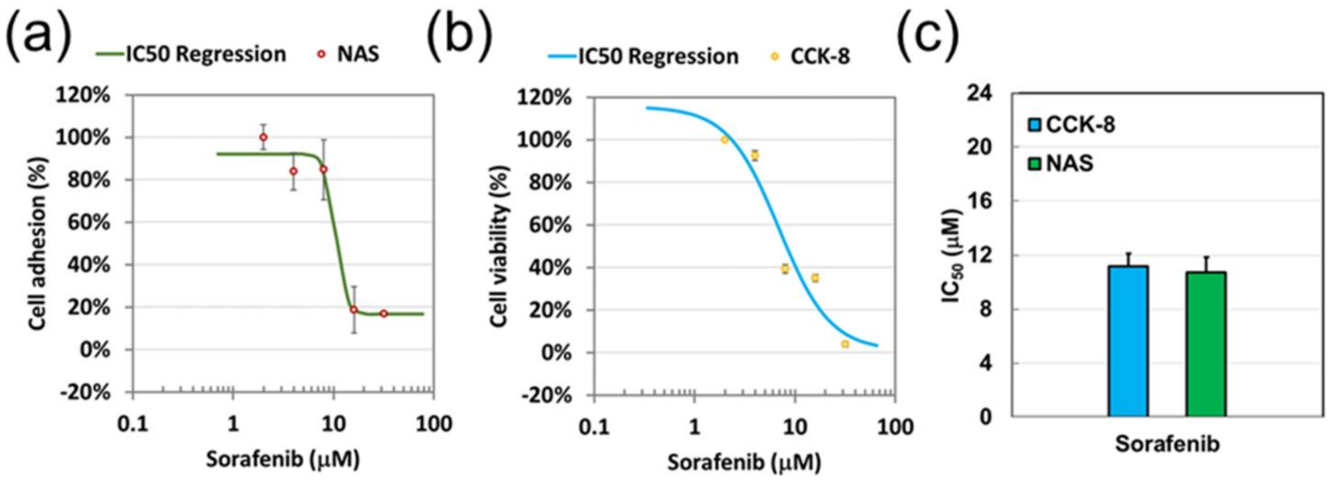

Figure S3. Effect of Sorafenib on cell viability and adhesion in Huh-7 cells. Cytotoxicity assessment with (a) NAS, (b) CCK-8. (c) Statistical results of IC_{50} values obtained from 4 independent tests of CCK-8 and NAS. The IC_{50} values were calculated with the AAT Bioquest calculator using a nonlinear regression curve fitting with a variable slope (four parameters)

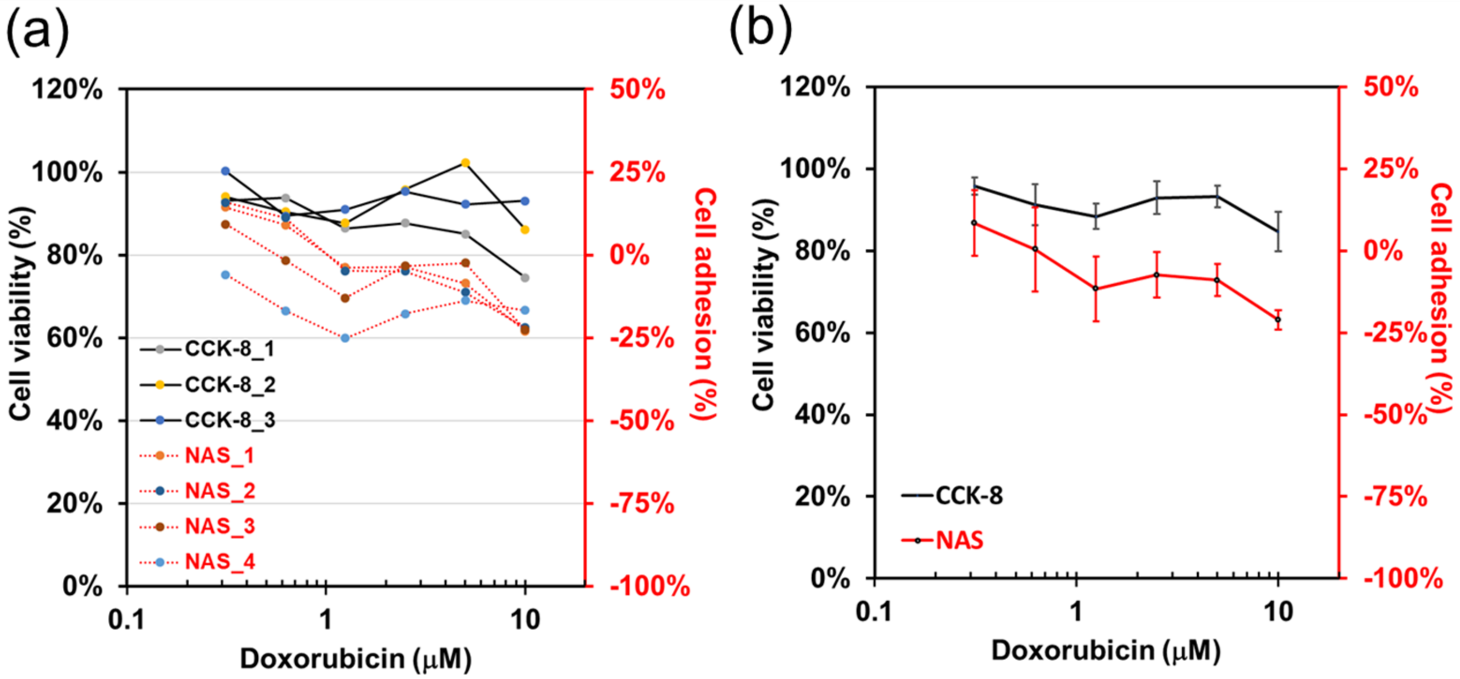

Figure S4. Effect of DOX on cell viability and adhesion assessed using NAS and CCK-8 in A549 cells. (a) Independent assessments using the CCK-8 assay for cell viability and the NAS assay for cell adhesion revealed consistent responses to DOX-induced cytotoxicity. Both methods showed a 20-35% reduction in cell survival or relative adhesion following treatment, indicating that 10 μM DOX does not exert significant cytotoxic effects on A549 cells. (b) Comparative statistical analysis between the two assays supports the presence of resistance to 10 μM DOX in A549 cells.

Table S1. Comparison of cell viability assays

System	Price per 100 Tests	96-Well Plate Required	Technique Skill Level	Mechanism	Reference Method	Company
**NAS**	US$33.33	Not required	Low	Cell adhesion	Self-reference in each test	–
**CCK-8**	US$95.40	Required	Low	Enzyme activity	Extra test needed for reference	ALD-Sigma
**Cell Staining**	US$147.00	Required	High	Membrane permeability	Extra test needed for reference	Beyotime

Table S2. A comparative analysis of the SPR-based cytotoxicity assessment technique with established electrical and optical sensing methods for evaluating cytotoxicity and determining IC_{50} values.

Sensing Modality	Cell Line	Drug	IC_{50}	Platform Details	Reference
SPR-based cytotoxicity assay	—	—	—	No reports to date of SPR angle or intensity shifts being used to derive an IC_{50} in cancer cells.	—
SERS-based intracellular probe	A549	Doxorubicin	—	Plasmonic nanoparticle tags enter cells, SERS peak intensity correlates with uptake	Fortuni et al., ACS Sensors 2023
Electrochemical (CV/DPV/EIS) cytosensor	MCF-7	Doxorubicin	—	Glassy carbon electrode modified with CNTs + AuNPs; cells immobilized in gelatin matrix; viability ↔ current calibration curve	Soltani Gohari et al., J. Mol. Liq. 2025
SPRi-based cytotoxicity assay	CL1-0<br>MCF-7<br>Huh-7	Doxorubicin<br>Doxorubicin<br>Sorafenib	3.03 μM<br>1.5 μM<br>10.7 μM	SPR imaging platform utilizing a plastic-based nanostructured gold film sensor	This study
